# Understanding Parenting Intentions Among Childfree Gay Men: A Comparison With Lesbian Women and Heterosexual Men and Women

**DOI:** 10.3389/fpsyg.2020.00430

**Published:** 2020-03-24

**Authors:** Joke T. van Houten, Samantha L. Tornello, Peter J. Hoffenaar, Henny M. W. Bos

**Affiliations:** ^1^Department of Child Development and Education, University of Amsterdam, Amsterdam, Netherlands; ^2^Human Development and Family Studies, Pennsylvania State University, University Park, PA, United States

**Keywords:** gay men, childfree, parenting intentions, theory of planned behavior, intended parents

## Abstract

**Introduction:**

There is a growing interest in the parenting intentions of gay men. Prior research has found that gay men are less likely to become parents compared to their heterosexual and lesbian peers, but we know very little about why this discrepancy exists. Our first aim was to investigate whether the strength of parenting intentions is similar or different among childfree gay men compared to lesbian women, and heterosexual men and women. Our second aim was to explore the extent to which the theory of planned behavior (TPB) model (attitude, subjective norms, and self-efficacy) is universal in predicting the strength of parenting intentions across gender and/or sexual orientation.

**Methods:**

The study was based on a United States cross-sectional, internet-based survey of childfree people who want to become parents in the future. The sample consisted of 58 gay men, 66 lesbian women, 164 heterosexual people (128 women and 36 men).

**Results:**

A Bayesian ANCOVA showed no support for a gender difference in the strength of parenting intentions. Moderate evidence was provided for gay men and lesbian women reporting a similar strength of parenting intentions compared to their heterosexual peers. Bayesian linear regression analyses showed that perceived positive and negative life changes were stronger predictors of the strength of parenting intentions for men than for women. Perceived positive life changes predicted the strength of parenting intentions similarily across sexual orientations. For gay men and lesbian women, perceived parental acceptance of future parenthood was a weaker predictor of the strength of parenting intentions compared to heterosexual people.

**Conclusion:**

Those who perceived parenthood as bringing positive life changes, especially for men, expressed stronger parenting intentions.

## Introduction

Becoming a parent is a universal desire for many young people (Purewal and Van den Akker, 2007), however, parenthood is not always possible for sexual minority people, especially gay men. Gay men who want to become parents experience a number of legal (Kazyak et al., 2018) and financial barriers (Smietana, 2018), along with greater experiences of stigmatization (e.g., Berkowitz and Marsiglio, 2007; Baiocco et al., 2012; Goldberg et al., 2012; Carone et al., 2017). In addition, gay men more often face greater complexities when deciding how to become a parent (surrogacy, adoption, co-parenting, and foster care; Murphy, 2013; Smietana et al., 2014; Carone et al., 2017; Smietana, 2018). Nevertheless, many gay men want to become parents in the future (Gates et al., 2007; Goldberg et al., 2012; Scandurra et al., 2019). Yet little is known about the decision-making process of childfree gay men toward becoming parents in the future (Mezey, 2013; Gato et al., 2017; Riskind and Tornello, 2017; Scandurra et al., 2019). The present study focuses on the parenting intentions of childfree gay men, compared to their lesbian and heterosexual peers.

For childfree gay men, there is a gap between future parenthood desires and intentions compared to heterosexual men. Riskind and Patterson (2010) examined parenting desires and intentions among a United States representative sample [2002 National Survey of Family Growth (NSFG)] and found that gay men (54%) were significantly less likely to desire future parenthood compared to their heterosexual peers (75%). Among men who desired future parenthood, gay men (75%) were significantly less likely to intend to become parents in the future compared to their heterosexual peers (90%; Riskind and Patterson, 2010). Riskind and Patterson (2010) found that for men, but not for women, sexual orientation was a significant predictor of future parenting intentions. In a replication a few years later (2011-2013), researchers found the same patterns among gay men, with gay men reporting lower parenthood desires and intentions compared to their heterosexual, bisexual, and lesbian peers (Riskind and Tornello, 2017). These findings have been replicated in a number of other countries, such as in Israel (Shenkman, 2012) and Italy (Baiocco and Laghi, 2013).

A theoretical model that is often used to understand the decision-making process of becoming a parent among childfree people is the theory of planned behavior (TPB). According to the TPB (Ajzen, 1991), an individual’s attitudes, subjective norms, and perceived behavioral control or self-efficacy (Bandura, 1997) are important factors in all decision-making processes. Previous studies have demonstrated the TPB could be useful in understanding parenting intentions generally not necessarily in terms of gender or sexual orientation (Ajzen and Klobas, 2013) and among gay and heterosexual men (Kranz et al., 2018). Based on the TPB, parenting intentions would be predicted by an individual’s perceived life changes of future parenthood (attitudes), along with his or her personal desire to conform to these social expectations (subjective norms), and perceived control or belief that he or she can become a parent in the future (self-efficacy; Ajzen and Klobas, 2013).

Previous studies using the TPB as a model to examine future parenthood have focused on whether or not people intend to become parents (Billari et al., 2009; Ajzen and Klobas, 2013; Kranz et al., 2018), both in the short term and longer term (Dommermuth et al., 2011). A Bulgarian representative study suggested that attitudes and subjective norms, but not perceived behavioral control, predict whether men and women intend to become parents within two years (Billari et al., 2009). Interestingly, subjective norms were found to be a stronger predictor of parental intentions among women than men. In addition, a Norwegian representative study suggested that subjective norms, but not attitudes, predicted short-term parenting intentions among childfree people, although self-efficacy was not measured in this study (Dommermuth et al., 2011).

There has been limited research using the TPB among sexual minority childfree people. In a study of childfree heterosexual and gay men researchers found that attitudes and perceived behavioral control, but not subjective norms, were strong predictors of future parenting intentions among men regardless of sexual orientation (Kranz et al., 2018). In this study, the perceived benefits and costs of parenthood (attitudes), the attitudes of others toward future parenthood (subjective norms), and parenthood self-efficacy were directly associated with fathering intentions of gay men and heterosexual men. These direct associations were significant, albeit weak. However, fathering desires meditated on the relationship between attitudes and fathering intentions and between self-efficacy and fathering intentions. For gay and heterosexual men attitudes and self-efficacy predicted fathering desires, and fathering desires in turn predicted fathering intentions. Despite the fact that gay men reported lower levels of self-efficacy and less acceptance from others compared to heterosexual men, there was no difference in the extent to which components of the TPB predicted parenting intentions for men regardless of sexual orientation.

Although these studies showed support for the TPB model regarding general parenting intentions, these studies did not examine the strength of these intentions. We know that gay (intended) fathers express a deep-rooted, strong desire to becoming parents (Gianino, 2008; May and Tenzek, 2016; Fantus and Newman, 2019). Due to their sexual minority status, gay men, like lesbian women, are highly exposed to stigma (Meyer et al., 2011) and receive less social support compared to heterosexual people (Frost et al., 2016), this is particularly true when it comes to gay parenthood among gay men (Berkowitz and Marsiglio, 2007). When gay men intend to fulfill their deeply rooted parenting desire, they venture traditional role patterns (Carneiro et al., 2017). Parenthood is regarded as the natural domain of women, with women often assumed of being the primary caregiver (Wells, 2011; Henderson et al., 2016). Gay men who plan to have a child may feel stigmatized that they would be below par as parents compared to women (Wells, 2011). It therefore seems likely that men who intend to fulfill their parenting desire are highly motivated as they have already experienced parenting related stigmas and other barriers. Additionally, gay men who already plan to have children might have similar determination regarding these intentions compared to women. With this in mind, there may be a conceptual difference between having or not having parenting intentions. In a study of gay and bisexual men and women researchers found no gender differences in the strength of parenting intentions (Costa and Bidell, 2017). However, this study did not use the TPB model as a predictive pathway, only explored the strength of the parenting intentions. No prior work, to date, has used the TPB model to examine the strength of parenting intentions across both sexual orientation and gender. Due to this gap in the research, it is unknown to what extent the TPB predicts the strength of parenting intentions among those who want to become parents in the future and whether this varies across both gender and sexual orientation.

In order to understand the relevance of the TPB across gender and sexual orientation among those who intend to becoming parents, the present study focuses on the strength of parenting intentions among childfree gay men, lesbian women, and heterosexual men and women who want to become parents in the future. Similar to Kranz et al. (2018), we combined a level-oriented (comparing variables across groups) with a structure-oriented approach (comparing associations across groups) to investigate whether associations between variables differed based on gender and sexual orientation. Unique to this study, we also investigated the TPB among lesbian and heterosexual women.

In some of the studies discussed, the effect of the TPB predictors has been examined in separate models for groups based on gender (see e.g., Billari et al., 2009) or sexual orientation (Kranz et al., 2018). Such an analytic strategy is limited in that the comparison of two effects should be accompanied by a report of the statistical significance of their difference (Nieuwenhuis et al., 2011). Others may have failed to determine the strength of evidence for the null or alternative hypothesis, but at least statistically compared the magnitude of effects. For example, Kranz et al. (2018) did not find a significant difference in the effect of the TPB components on fathering intentions between gay and heterosexual men using equality constraints in a SEM model, although, they did not test for similarity. We along with Sakaluk (2019) note that their use of frequentist statistics did not allow for a conclusion that the evidence favors the hypothesis that sexual orientation is not a factor (for more information see the fallacy of negative proof: absence of evidence is not evidence of absence). In general, only calculating classical frequentist *p*-values seems ill-suited to determine whether groups based on gender and sexual orientation show similarities or differences in parenting intentions. We therefore followed recent recommendations made by Sakaluk (2019) to use Bayes Factors to also test similarities in groups, because according to the gender similarities hypothesis (Hyde, 2005) it is a misconception that groups differ mainly on psychological variables and predictive pathways across gender and sexual orientation (Sakaluk, 2019).

The first aim of this study was to examine whether the strength of parenting intentions was the same across gender and/or sexual orientation. Although prior research has found that gay men are less likely to intend to become parents, those studies included gay men regardless of whether they believed they would become parents in the future. Due to the focus of the present study being on the magnitude of parenting intentions, we do not expect childfree gay men to report lower parenting intentions compared to lesbian women and heterosexual men and women. The second aim of the study was to explore the extent to which the TPB model (attitude, subjective norms, and self-efficacy) is universal or varies based on gender and/or sexual orientation, in predicting the strength of parenting intentions. We hypothesized that the TPB-predictors of attitude and self-efficacy regarding future parenthood, but not subjective norms, would be universal for childfree gay men, lesbian women, and heterosexual men and women in predicting the strength of parenting intentions. Prior research has found no differences in the extent to which attitudes predict the strength of parenting intentions among men and women (Billari et al., 2009). In addition, as with the study by Kranz et al. (2018), we expected components of the TPB to predict the strength of parenting intentions for gay men and heterosexual men to a similar extent. In contrast to the hypothesis regarding self-efficacy and attitudes, we hypothesized that the association between subjective norms and the strength of parenting intentions would be weaker for gay men and lesbian women compared to their heterosexual peers. As sexual minority people, gay men, like lesbian women, often have prior exposure to stigma (Meyer et al., 2011) and lack of social support (Frost et al., 2016), especially when it comes to gay parenthood (Berkowitz and Marsiglio, 2007).

## Materials and Methods

### Participants and Procedure

The study sample consisted of 288 childfree gay, lesbian, and heterosexual intended parents (cisgender women and men) who participated in 2015 in an internet-based study. Participants were recruited through targeted advertisements on social media and search engines. People who were interested in participating would contact the PI (Second Author), and if eligible to participate, they would receive a personalized password-protected link to the online consent form and survey. At the time of survey completion participants were provided the option to enter a raffle for 1 out of 24 twenty-five-dollar gift cards for Target stores. Participation in this study was voluntary and was approved by the Institutional Research Board of the Pennsylvania State University.

Since the focus of this study was on childfree cisgender gay men, lesbian women, and heterosexual men and women in the US who intended to become parents in the future, we excluded participants based on specific criteria. Of the 582 completed surveys, in order to preserve data independence, only one member of a couple participated (*n* = 43), we removed all participants who did not currently reside in the US (*n* = 67), who did not identify their sexual orientation as heterosexual, lesbian, or gay (*n* = 160), not identifying themselves or their partner as cisgender (*n* = 19), were in a polyamorous relationship (*n* = 1), and described their ideal number of children as zero (*n* = 4) resulting in a final sample of 288 self-identified childfree intended parents. Gender and sexual orientation breakdown of the sample was as follows: 58 gay men (20.1%), 66 lesbian women (22.9%), 36 heterosexual men (12.5%), and 128 heterosexual women (44.4%). Participants were 18 to 52 years old (*M* = 27.82, *SD* = 5.87). Most participants self-identified as White/European American (79.9%), reported receiving a bachelor’s degree or higher (64.6%), and worked an average of 32.83 h per week in paid employment (*SD* = 17.26). The majority of the participants were in a committed relationship (74.7%) for an average for 5.17 years.

A few significant group differences in demographic characteristics were found. Gay men were significantly less likely to identify as White/European American (62%) compared to the other groups [82–91%; *X*^2^ (3) = 15.80, *p* < 0.01]. In addition, gay men were more likely to be single (65%) compared to lesbian women (18%), and heterosexual men (6%) and women [16%; *X*^2^ (3) = 64.11, *p* < 0.001]. A one-way analysis of variance (ANOVA) for educational level showed a significant gender by sexual orientation interaction [*F*(1,284) = 3.92, *p* = 0.05], suggesting group differences.

### Measures

#### Demographics

Participants were asked to provide demographic details about themselves and their partner (if applicable). Information included age, gender, race/ethnic identity, sexual orientation, educational attainment, hours per week in paid employment, relationship status, and relationship length.

#### Strength of Parenting Intentions

One single item (Van Balen and Trimbos-Kemper, 1995) measured the strength of the intentions to become a parent: “What are you willing to give up to have children?” (1 = *it does not matter whether or not I become a parent* to 6 = *I will do everything to become a parent*). A high score on this item indicated stronger intentions to become a parent.

#### Attitudes

Beliefs about emotional benefits of parenthood were measured using *Idealization of parenthood*, an 8-item scale (Eibach and Mock, 2011). In order to obtain a good reliability of the scale, the 3 negatively formulated items, which had a negative influence on the reliability after recoding, were excluded, leaving a 5-item scale (α = 0.82). Items for this measure included “*Parents experience a lot more happiness and satisfaction in their lives compared to people who have never had children*” and “*There is nothing more rewarding in this life than raising a child*” (−2 = *strongly disagree* to 2 = *strongly agree*). Scores of the 5 items were summed, with higher scores indicating a stronger belief that parenthood offers emotional benefits.

Expected possible consequences of parenthood were measured using *Perceived life changes in connection with becoming a parent*, a 14 item-scale (Lampic et al., 2006). Participants were asked to what extent they agreed with possible consequences of future parenthood. Responses were measured using a 5-point Likert scale (1 = *disagree* to 5 = *entirely agree*). To be able to distinguish between positive and negative expectations, we divided this scale into two subscales: *perceived positive life changes* and *perceived negative life changes.* The *perceived positive life changes* contained 9 items, including “*I will develop as a person*” and “*Everyday life will be more enjoyable.*” This scale had good reliability (α = 0.80). The *perceived negative life changes* included 5 items, like “*Less time to devote to work and a career*” and “*Less time for my own interests.*” This scale had sufficient reliability (α = 0.77). A total score was calculated for each sub-scale, with higher scores indicating more positive or negative (respectively) expectations of future parenthood.

#### Family Acceptance

Participants were asked a series of questions regarding family members’ acceptance of potential future parenthood. Participants answered the question “How accepting are the people below regarding your wish to become a parent?” for their parents, siblings, and extended family members (0 = *not accepting at all* to 5 = *fully accepting*). Due to the data being highly skewed (among heterosexual men, the values 0 and 1 did not occur for parental acceptance), we dummy recoded this variable with participant responses of 1 thru 4 to 0 (*not accepting*) and 5 to 1 (*accepting*).

#### Self-Efficacy

Participant’s self-efficacy regarding future parenthood was measured using the *Parenting Competence scale* (Johnston and Mash, 1989). This scale consisted of 7 items, for example “*I think that being a parent is manageable*, *and any problems are easily solved*” and “*I think I will meet my personal expectations for expertise in caring for my baby,”* and were answered using a 1 (*strongly disagree*) to 6 (*strongly agree*) scale. Scores were summed, with higher scores indicating a higher level of self-efficacy. This scale had good reliability (α = 0.82).

### Inferential Statistics

The data analysis was carried out using the JASP software version 0.8.6.0 (JASP Team, 2020). This program offers standard statistical procedures in Bayesian form. Because traditional forms of null hypothesis significance testing do not allow one to determine the relative strength of the evidence for a null or alternative hypothesis, they seem ill-suited to determine whether groups based on gender and sexual orientation show similarities or differences in parenting intentions (Sakaluk, 2019). Similarly, null hypothesis significance testing might be appropriate when anticipating differences among sexuality-related groups in the relative explanatory power of attitudes, norms and control, but it is not possible to infer equivalence of regression slopes bases on non-significant interaction effects. In order to address the question whether TPB factors are universal or specific in predicting the strength of parenting intentions among childfree intended gay, lesbian and heterosexual intended parents, we chose to test with Bayesian alternatives. More specifically, we used the Bayes factor (BFs; Rouder et al., 2018). The BF indicates whether the data would be more likely under an alternative hypothesis (group difference or differential effects) than under the null hypothesis (equivalence or invariance). Generally, BFs greater than three are taken as evidence in favor of the alternative over the null hypothesis (BF_10_) or in favor of the null over the alternative hypothesis (BF_01_). Bayes Factors below the threshold of 3 were interpreted as representing weak evidence. In a Bayesian perspective, weak (or anecdotal) evidence indicates that we hesitate or are reluctant to change our beliefs based on the difference between what we predicted and what we observed (Jarosz and Wiley, 2014). Alternatively, weak evidence can make one decide that there was not enough information to make a conclusive decision in favor of the null or alternative hypothesis.

## Results

### Strength of Parenting Intentions

**FIGURE 1:**
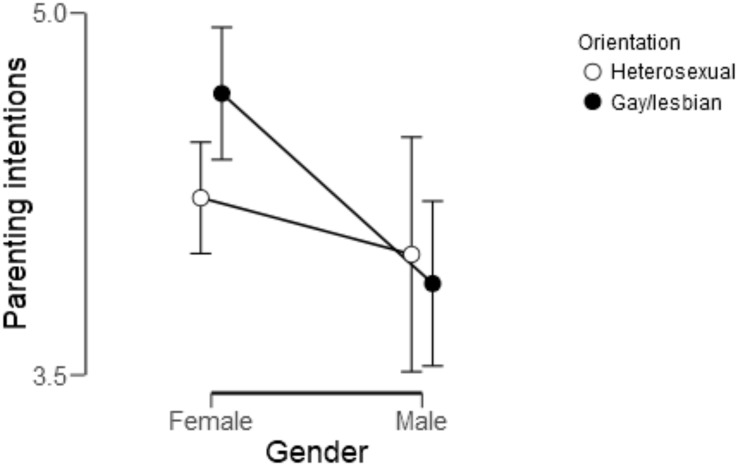
The strength of parenting intentions among gay men, lesbian women and heterosexual men and women. Points represent group averages, *n* = 281. Responses were on 6-point Likert scale with higher scores indicating a stronger intent to become a parent.

Using Bayesian versions of a 2 (men vs. women) × 2 (gay and lesbian participants (men and women) vs. heterosexual participants) ANCOVA, we tested whether the strength of parenting intentions differed between childfree gay, lesbian and heterosexual intended parents. Due to significant differences in race/ethnicity, educational attainment, and relationship status, these demographic variables were included as covariates. In the Bayesian ANCOVA, two models including main effects of gender or sexual orientation, a model with both main effects and a model with both main effects and an interaction effect were compared against the null model, which only contained the set of control variables (race/ethnicity, educational attainment, and relationship status). The default JASP priors for fixed effects were used. Bayesian model comparison revealed that the model with only the main effect of gender was the best model. Women scored higher on the strength of parenting intentions (*M* = 4.32, *SD* = 0.13) than men (*M* = 3.90, *SD* = 0.15), see Figure 1. The support for favoring the model with only the main effect of gender over the null model was weak (BF_10_ = 1.29), meaning that the data were 1.29 times more likely to be observed under the alternative hypothesis (gender difference) than under the null hypothesis (similarity across groups) and that it is not possible to falsify the gender similarities hypothesis. With regard to sexual orientation, the Bayesian model comparison showed moderate evidence for similarity across groups (BF_01_ = 3,45), which means that the data were more than 3.45 times less likely under the alternative hypothesis (sexual orientation differences) than under the null hypothesis (similarity across groups).

### TPB-Predictors of Parenting Intentions

Using Bayesian linear regression analyses, we explored to what extend the TPB-predictors: (1) attitudes (idealization of parenthood, perceived positive and negative life changes in connection with becoming a parent), (2) subjective norms (acceptance of parents, siblings, and extended family members), and (3) self-efficacy were universal or different for childfree gay, lesbian and heterosexual intended parents in predicting the strength of parenting intentions. In the Bayesian linear regression analyses, two models including interaction effects of gender or sexual orientation on the TPB predictor, and a model with both interaction effects of gender and sexual orientation on a TPB predictor were compared against the null model, which contained the set of control variables (race/ethnicity, relationship status, and educational attainment) and the variables gender, sexual orientation and a TPB predictor. The default JASP priors for fixed effects were used.

#### Attitudes

**FIGURE 2:**
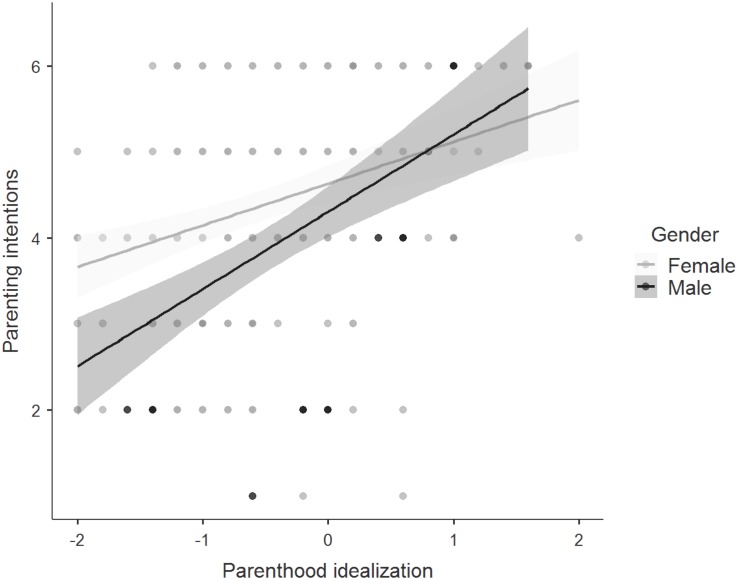
Regression plot showing the relationship between idealization of parenthood and the strength of parenting intentions among men and women. Points represent individual responses, *n* = 264 and the gray shaded region represents the 95% confidence region. For idealization, responses were on 5-point Likert scale with higher scores indicating a stronger belief that parenthood offers emotional benefits. For the strength of parenting intentions, responses were on 6-point Likert scale with higher scores indicating a stronger intent to become a parent.

Although model comparisons showed that a regression model including an interaction effect between gender and idealization was the best model, Bayesian analysis indicated weak evidence for an interaction effect between gender and idealization of future parenthood (BF_10_ = 1.72), see Figure 2. The data were 1.72 times more likely to be observed under the alternative hypothesis (gender difference) than under the null hypothesis (similarity across groups). Adding the interaction effects increased the variance explained from 20 to 22%. With regard to sexual orientation, the Bayesian model comparison showed weak evidence for similarity across groups (BF_01_ = 2.50). The data were 2.5 times more likely under the null hypothesis compared to the alternative hypothesis.

**FIGURE 3:**
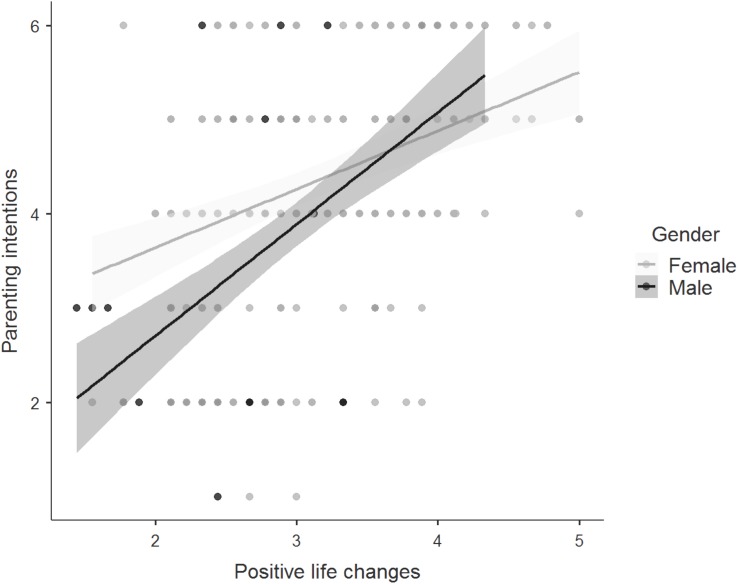
Regression plot showing the relationship between perceived positive life changes in connection with becoming a parent and the strength of parenting intentions among men and women. Points represent individual responses, *n* = 269 and the gray shaded region represents the 95% confidence region. For perceived positive life changes, responses were on 5-point Likert scale with higher scores indicating more positive expectations of future parenthood. For the strength of parenting intentions, responses were on 6-point Likert scale with higher scores indicating a stronger intent to become a parent.

The association between perceived positive life changes in connection with becoming a parent and the strength of parenting intentions was greater for men than for women (see Figure 3). The model comparison showed that the model with only an interaction effect between gender and perceived positive life changes was the best model, providing moderately stronger evidence in favor of the model including the interaction against the null model (BF_10_ = 9.97). The data were 9.97 times more likely to be observed under the alternative hypothesis (gender differences) than under the null hypothesis (similarity across groups). The amount of variance explained increased from 25% to 27% by including the interaction effects. Next to this, Bayesian analysis indicated moderate evidence in favor of invariance across sexual orientation, i.e., the null model against a model including the interaction effect (BF_01_ = 3.72). The data were 3.72 times less likely under the alternative hypothesis compared to the null hypothesis.

**FIGURE 4:**
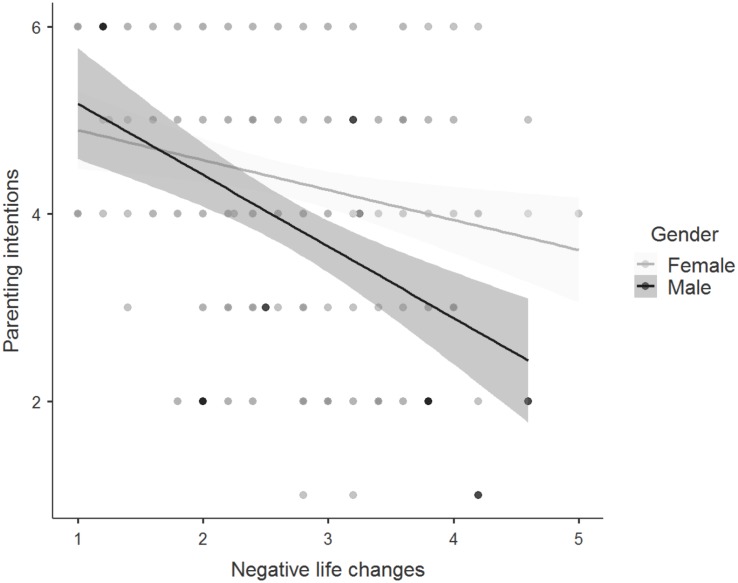
Regression plot showing the relationship between perceived negative life changes in connection with becoming a parent and the strength of parenting intentions among men and women. Points represent individual responses, *n* = 269, and the gray shaded region represents the 95% confidence region. For perceived negative life changes, responses were on 5-point Likert scale with higher scores indicating more negative expectations of future parenthood. For the strength of parenting intentions, responses were on 6-point Likert scale with higher scores indicating a stronger intent to become a parent.

The association between perceived negative life changes in connection with becoming a parent and the strength of parenting intentions was also greater for men than for women (see Figure 4). The Bayesian model comparison revealed moderate evidence that the model with an interaction effect between gender and perceived negative life changes was the best model and had to be preferred over the null model (BF_10_ = 3.78). The data were 3.78 times as likely under the alternative hypothesis than under the null hypothesis. Including the interaction effects increased the variance explained from 12 to 14%. Weak evidence was shown for sexual orientation similarity across groups (BF_01_ = 2.34). The data were 2.34 times as likely under the null hypothesis.

#### Subjective Norms

**FIGURE 5:**
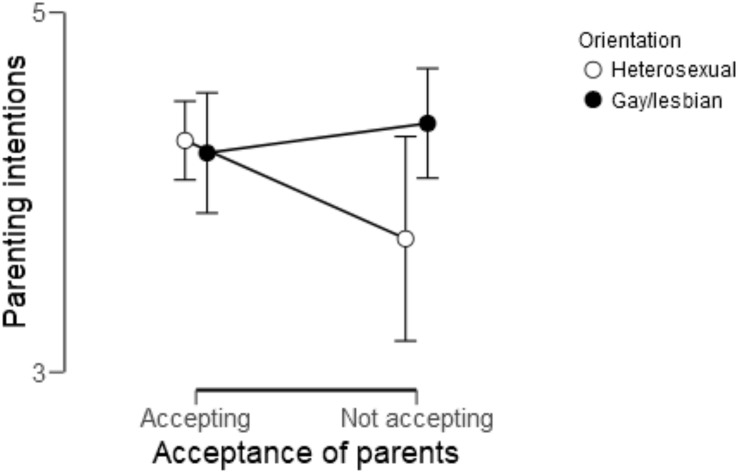
Regression plot showing the relationship between the acceptance of parents regarding potential future parenthood and the strength of parenting intentions among gay men and lesbian women vs. heterosexual men and women. Points represent group averages, *n* = 287. Acceptance of parents was dummy recoded with responses of 1 thru 4 to 0 (*not accepting*) and 5 to 1 (*accepting*). For the strength of parenting intentions, responses were on 6-point Likert scale with higher scores indicating a stronger intent to become a parent.

The association between the acceptance of parents regarding potential future parenthood and the strength of parenting intentions was weaker for lesbian women and gay men compared to heterosexual women and men (see Figure 5). The Bayesian model comparison provided moderate evidence that the model with an interaction effect of sexual orientation on acceptance of parents was the best model (BF_10_ = 4.91). It was 4.91 as likely to find the data under the alternative hypothesis than under the null hypothesis. The variance explained increased by 1% by adding the interaction effects with the total variance explained becoming 9%. The analyses provided weak support for gender similarity across groups (BF_01_ = 2.61). The data were 2.61 times as likely under the null hypothesis. With regard to the associations between the acceptance of siblings or extended family members and the strength of parenting intentions, the null model was the best model (BF_10_ = 1.00), which means that no evidence was found to verify or falsify the gender similarities hypothesis.

#### Self-Efficacy

**FIGURE 6:**
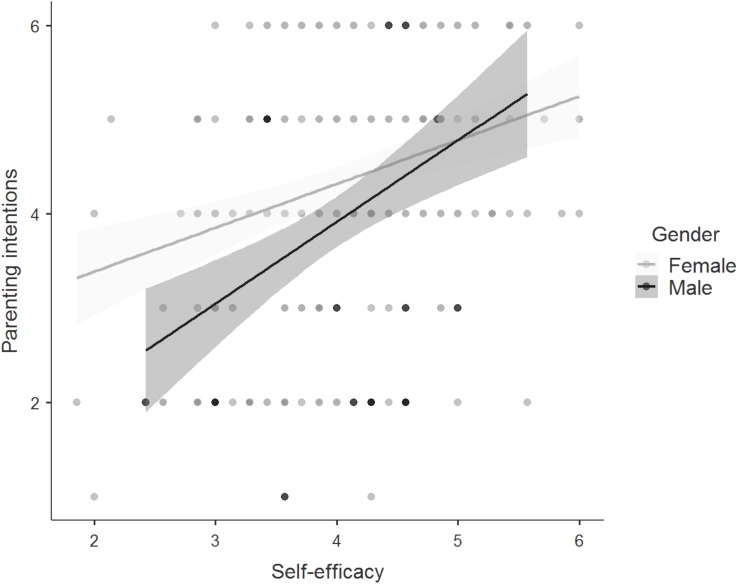
Regression plot showing the relationship between self-efficacy regarding future parenthood and the strength of parenting intentions among men and women. Points represent individual responses, *n* = 262, and the gray shaded region represents the 95% confidence region. Responses were on 6-point Likert scales. Higher scores on self-efficacy indicated a higher level of self-efficacy regarding future parenthood. Higher scores on the strength of parenting intentions indicated a stronger intent to become a parent.

Bayesian analyses yielded the model with an interaction effect of gender on self-efficacy to be the best model, suggesting the association between self-efficacy and the strength of parenting intentions was the strongest for men (see Figure 6). Support for this finding was weak (BF_10_ = 1.27), which means that the data were 1.27 times less likely to be observed under the alternative hypothesis (gender differences) than under the null hypothesis (similarity across groups). Adding the interaction effects increased the variance explained from 17 to 18%. Weak evidence was shown for sexual orientation similarity across groups (BF_01_ = 1.88). The data were 1.88 times as likely under the null hypothesis.

## Discussion

The first aim of this study was to gain insight into whether the strength of parenting intentions was similar or different across the groups as a function of gender and sexual orientation. In line with our expectations, no support was found that men would be less willing to give up different aspects of their lives to have children compared to women. Also in line with our expectations, sexual orientation was not a predictor of the strength of parenting intentions. Gay men and lesbian women expressed a similar strength of parenting intentions compared to their heterosexual peers.

The similarity in the strength of parenting intentions among lesbian women and gay men and heterosexual people is not in line with prior research that has found that gay men express less often the intention to have a child compared to heterosexual men (e.g., Riskind and Patterson, 2010; Shenkman, 2012; Baiocco and Laghi, 2013; Riskind and Tornello, 2017). This discrepancy might support the premise of this study that there is a conceptual difference between having or not having parenting intentions. Given the experienced barriers to becoming parents (Baiocco et al., 2012; Kazyak et al., 2018; Smietana, 2018), it is plausible that gay men do not convert their desire to have a child into parenting intentions as often as heterosexual men do. Although, once gay men plan to have children, they seem to have experienced a change in their procreative consciousness and see opportunities to overcome barriers and to fulfill their desire to have children (Smietana, 2018). As a consequence, gay men seem to be willing to give up as much as heterosexual men in order to fulfill their desire to have children.

To the best of our knowledge, this was the first study to explore whether TPB-predictors attitude, subjective norms and self-efficacy are universal or specific for childfree gay, lesbian and heterosexual intended parents, in predicting the strength of parenting intentions, which was the second aim of the present study. Overall, the analyses often gave a similar picture across groups, although, some important group differences were found. Contrary to our expectations and previous research (Billari et al., 2009), we found two meaningful effects of gender. For men, both expected positive and negative life changes in connection with becoming a parent were stronger predictors of the strength of parenting intentions compared to women. These gender differences might be reflective of the heteronormative perspective on parenthood. From this perspective, women are expected to become mothers and primary caregiver but expectations for men about the parental role are often different (Wells, 2011; Henderson et al., 2016). As a result, intrinsic motivations like expected life changes in connection with becoming a parent might be more important for man than for women. According to our findings those who showed stronger parenting intentions also saw greater positive life changes and less negative life changes. This was particularly true for men.

In line with our expectations and previous research that has found that stigmatization of sexual minorities undermines feelings of being accepted (Meyer et al., 2011), the acceptance of parents regarding potential future parenthood was a stronger predictor of the strength of parenting intentions for heterosexual people than for gay men and lesbian women. Contrary to our expectations, no gender effect was found on subjective norms. These findings might reflect the well-developed gay identity, along with a future parent identity of the gay men in this study with all having intentions to become parents in the future. Gay men who plan parenthood have to deal with hardships like biological (Mezey, 2013), financial (Smietana, 2018), legal barriers (Kazyak et al., 2018), and internalized and externalized stigmas because they belong to a sexual minority status and challenge traditional parenting patterns (e.g., Goldberg et al., 2012; Carneiro et al., 2017). In facing these hardships, gay men who intend to become fathers generally lack a role model of a father being gay and being the primary caregiver, coping with similar hardships (Gianino, 2008). As a consequence, gay men planning to become parents in the future have to reconsider their meaning of fatherhood and think about their identity in the context of parenthood (Shenkman and Shmotkin, 2016). Prior research among gay fathers has found that in the process of planning parenthood, gay men were able conquer negative stereotypes about gay fathers (Gianino, 2008). Despite all these barriers, the gay men who participated in the current study intended to have children and their parenting intentions were not weaker than lesbian women.

In line with our expectations and the gender similarities hypothesis (Hyde, 2005), no support was found for the TPB factor self-efficacy to be a stronger predictor for men or for women in predicting the strength of parenting intentions. In line with prior research (Kranz et al., 2018), no difference based on sexual orientation was found. The extent to which self-efficacy predicted the strength of parenting intentions did not differ between gay men and their heterosexual peers. However, contrary to our expectations, no support was found that self-efficacy predicted the strength of parenting intentions to the same extent for gay men and heterosexual men.

Noteworthy, the TPB predictors were not equally relevant in predicting the strength of parenting intentions. Consistent with previous research among men (Kranz et al., 2018), the intrinsic motivational TPB predictors attitudes and self-efficacy were more relevant in predicting the strength of parenting intentions than the more extrinsic predictor subjective norms. The attitude component expecting positive life changes in connection with becoming a parent was the most relevant predictor in the TPB model, explaining 27% of the variance in the strength of parenting intentions. The subjective norms component (acceptance of parents) regarding potential future parenthood explained no more than 9% of the variance in people’s strength of parenthood intention did not seem to predict the strength of parenting intentions.

Certain limitations of this study should be taken into account. First of all, the current study focused on TPB-factors to understand the decision-making process of becoming a parent among childfree gay men. There could be a number of other relevant factors in this decision-making process like internalized and externalized stigmas due to the sexual minority status of gay men (Goldberg et al., 2012; Carone et al., 2017). We recommend that future research take these factors related to minority stress into account. Such research can be embedded in the theoretical framework of the minority stress theory (see Meyer, 2003). Secondly, we only included people in the sample who intended to have children in the future. Those who had no parenting intentions were not part of this study. Therefore, the current study does not provide any insight into predicting who will or will not have parental intentions. The purpose of the study was to gain insight into differences in and predictors of the strength of parenting intentions of those who already intend to become. Thirdly, only cisgender gay men, lesbian women and heterosexual men and women were included in the study. Future research should also include bisexual or gender minority people. Fourthly, this study did not take into account the role of partners in the participants’ parenting intentions, which is important to address since the decision to become a parent is often made at a couple level rather than on an individual level (Shreffler et al., 2017). Partners could influence the parenting intentions of each other, similarly to the findings that partners can influence each other in how they think about internalized stigmas (Goldberg et al., 2012). Nevertheless, based on the TPB, we were interested in predictors of the strength of parenthood intention at the individual level. Therefore, the couple level was not taken into account in the current study. Further research is needed in order to gain insight into the extent to which partners reinforce each other’s strength of parenthood intention and to determine the extent to which partners affect the TPB model for childfree gay, lesbian, and heterosexual intended parents. Finally, it should be mentioned that the strength of parenting intentions was measured with a single item as was done in prior research (e.g., Van Balen and Trimbos-Kemper, 1995; Bos et al., 2003) and is common when measuring parenting intentions (e.g., Riskind and Patterson, 2010; Riskind and Tornello, 2017). In addition, a study on the validity of single-item life satisfaction measures showed that single items provided almost equal information compared to a multiple-item scale (Cheung and Lucas, 2014).

This study was unique in that it examined not only differences but also similarities based on gender and/or sexual orientation, using statistical analyses not used in previous research in predicting the strength of parenting intentions. This study showed that the strength of parenting intentions was similar across groups based on sexual orientation. Gay men expressed a similar strength compared to their heterosexual peers. In predicting the strength of parenting intentions, the attitudes regarding future parenthood were the most relevant TPB predictor of the strength of parenting intentions. Those who expressed stronger parenting intentions, expected more positive life changes. This was similar across groups based on sexual orientation but was different based on gender. The extent to which positive life changes predicted the strength of parenting intentions was stronger for men compared to women. In addition, the stronger the intention to become parents, the less negative life changes men and women expected from becoming parents. This was also particularly true for men. Finally, this study showed moderate evidence for a difference based on sexual orientation. Although the TPB predictor subjective norms was not a strong predictor of the strength of parenting intentions, the acceptance of parents regarding future parenthood predicted to a greater extent the parenting intentions of heterosexual people than of gay men and lesbian women.

Overall, the TPB model seemed not to differ much across groups based on sexual orientation in predicting the strength of parenthood intention. However, the possibilities for gay and lesbian couples to convert their parenting intentions into behavior that can result in parenthood are not the same compared to their heterosexual peers (Riskind and Patterson, 2010; Riskind and Tornello, 2017). If the intention and underlying factors are largely the same for intended parent regardless of sexual orientation, law, and policy makers should make all pathways to becoming parents equally accessible to sexual minority people. When counseling gay men and lesbian women, reproductive health-care professionals should discuss how to arrange support during and after the transition to parenthood, because gay men and lesbian women cannot always count on acceptance and support from their own parents. In addition, men who intend to become parents have to overcome a number of obstacles to make these intentions a reality. Importantly, reproductive health professionals and adoption agencies should pay special attention to men, when it comes to the benefits and costs of future parenthood. Men in need of reproductive assistance have to overcome a number of obstacles to become parents. Assisting these men in keeping the benefits of future parenthood in mind could help support and motivate them to become fathers.

## Data Availability Statement

The datasets analyzed in this article are not publicly available because it is an ongoing study. Requests to access the datasets should be directed to ST at SLT35@psu.edu.

## Ethics Statement

The studies involving human participants were reviewed and approved by the Institutional Research Board of the Pennsylvania State University. Participants read consent and checked that they had informed consent before participating in the study.

## Author Contributions

ST was responsible for recruiting the participants as Principal Investigator and assisted editing the manuscript. HB was Co-investigator. JH took the lead in writing the article and was responsible for the method of analysis. PH gave advice on the method of analysis and conducted the statistical analyses. All authors made substantial intellectual contributions to the work, revised the manuscript, and approved it for publication.

## Conflict of Interest

The authors declare that the research was conducted in the absence of any commercial or financial relationships that could be construed as a potential conflict of interest.
